# Elevated IgM levels as a marker for a unique phenotype in patients with Ataxia telangiectasia

**DOI:** 10.1186/s12887-018-1156-1

**Published:** 2018-06-04

**Authors:** Alexander Krauthammer, Avishay Lahad, Lior Goldberg, Ifat Sarouk, Batia Weiss, Raz Somech, Michalle Soudack, Itai M. Pessach

**Affiliations:** 1 Department of Pediatrics, The Edmond and Lily Safra Children’s Hospital, 52625 Tel- Hashomer, Israel; 2 Pediatric Gastroenterology Unit, The Edmond and Lily Safra Children’s Hospital, Tel- Hashomer, Israel; 3 Pediatric Pulmonary Unit, The Edmond and Lily Safra Children’s Hospital, Tel- Hashomer, Israel; 4 Pediatric Radiology Unit, The Edmond and Lily Safra Children’s Hospital, Tel- Hashomer, Israel; 5 The Claudio Cohen Department of Pediatric Intensive Care, The Edmond and Lily Safra Children’s Hospital, Tel- Hashomer, Israel; 6 Sackler Faculty of Medicine, Tel-Aviv University, Tel-Aviv, Israel

**Keywords:** AT, Class switching, Complications, Elevated IgM, Immunoglobulins, Lung functions, Phenotype

## Abstract

**Background:**

Ataxia telangiectasia (AT) is a rare, multi-systemic, genetic disorder. Mutations in the ATM gene cause dysfunction in cell-cycle, apoptosis and V (D) J recombination leading to neurodegeneration, cellular, humoral immunodeficiencies and predisposition to malignancies. Previous studies have suggested that a sub-group of AT patients with elevated IgM levels have a distinct and more severe phenotype. In the current study we aimed to better characterize this group of patients.

**Methods:**

We performed a retrospective review of 46 patient records, followed from January 1986 to January 2015 at the Israeli National AT Center. Demographic, clinical, radiological, laboratory data was reviewed and compared between AT patients with elevated IgM levels (EIgM) and patients with normal IgM levels (NIgM).

**Results:**

15/46(32.6%) patients had significantly elevated IgM levels. This group had a unique phenotype characterized mainly by increased risk of infection and early mortality. Colonization of lower respiratory tract with *Mycobacterium gordonae* and *Pseudomonas aeruginosa* as well as viral skin infections were more frequent in EIgM patients. Patients with NIgM had a significantly longer survival as compared to patients with EIgM but had an increased incidence of fatty liver or cirrhosis. T-cell recombination excision circles and kappa-deleting element recombination circle levels were significantly lower in the EIgM group, suggesting an abnormal class switching in this group.

**Conclusions:**

EIgM in AT patients are indicative of a more severe phenotype that probably results from a specific immune dysfunction. EIgM in AT should be considered a unique AT phenotype that may require different management.

**Electronic supplementary material:**

The online version of this article (10.1186/s12887-018-1156-1) contains supplementary material, which is available to authorized users.

## Background

Ataxia telangiectasia (AT) is a rare multi-systemic disorder. Mutations in the ATM gene causes dysfunction in cell cycle, apoptosis, DNA repair and V (D) J recombination [1–3]. Neurodegeneration, predisposition to malignancies as well as cellular and humoral immunodeficiency are the main complications [4]. Patients that have no ATM kinase activity are highly affected, while those with residual kinase activity usually present less severe neurologic disabilities and milder immune deficiency [5–7]. Immunodeficiency is present in up to 70% of AT patients. Embryonic or absent thymus, premature ageing of the immune system, abnormal T and B cell neogenesis and abnormal surface T cell receptor expression were shown to play a crucial role in AT associated immunodeficiency [2, 8]. The humoral immunodeficiency in AT includes IgG2 and IgG4 deficiencies, low or absent IgA and IgE and abnormal humoral response to vaccination [2]. Cellular immunodeficiency includes leukopenia, low CD4 + T cell counts and impaired lymphoprolifirative responses to antigens and mitogens [9]. AT patients often suffer from recurrent lower respiratory tract infections with subsequent pulmonary insufficiency resulting from a combination of immunodeficiency, recurrent aspirations, decreased mobilization and abnormal muscle tone [3, 10, 11]. Poor growth, liver abnormalities and insulin resistant diabetes are also a part of the AT phenotype [12–14].

Several reports have suggested that a subgroup of patients with AT present with higher levels of IgM that might be associated with a more severe phenotype [15–19]. A more recent study describing a cohort of 61 patients, concluded that patients with Hyper IgM phenotype and IgG2 deficiency showed decreased survival compared to AT patients with NIgM [20].

The aim of the present study was to better characterize the subgroup of AT patients with EIgM. We hypothesized that patients with elevated IgM levels may represent a separate phenotype similar to that seen in Hyper IgM syndrome [21–23], leading to more severe infectious, neoplastic and pulmonary morbidity and eventually shorter survival.

## Methods

### Patients

The Israeli National Clinical Center for Ataxia Telangiectasia at the Edmond and Lily Safra Children’s Hospital, Sheba Medical Center cares for most of the Ataxia Telangiectasia patients in Israel. We retrospectively reviewed the medical records of 53 AT patients followed at the Center between January 1st, 1986 and January 31, 2015. Forty-six patients were enrolled in this study, and 7 were excluded due to lack of sufficient data. The diagnosis of AT was based on a well-established set of criteria, as previously published [14]. Briefly, in addition to a distinctive clinical presentation suggestive of AT one of the following was required: mutations in the *ATM* gene, homozygous or compound heterozygous (*N* = 34), abnormal signal of the *ATM* protein on western blot (*N* = 12), or immune deficiency/chromosomal breakage/T cell malignancy in the presence of increased α-fetoprotein and cerebellar atrophy on magnetic resonance imaging (MRI) (*N* = 3). A specialized multidisciplinary team including an immunologist, pulmonologist and gastroenterologist evaluated all the patients every 6 month, or more frequently if required.

Patients with IgM levels at least 20% higher than the upper normal limit (Additional file 1: Table S1), on at least two separated occasions, were categorized as “elevated IgM” (EIgM). Clinical characteristics, disease course and complications were compared between EIgM patients and patients with NIgM. Immune system function, pulmonary function, infections, liver enzymes and predisposition to cancer were analyzed and results were compared between the two groups.

The study was approved by the Sheba Medical Center Institutional review board (SMC-IRB). The data retrieved from all patient charts was coded and de identified prior to analysis, hence the need for written consent was formally waved by the SMC-IRB.

### Infections and immune function

We retrospectively reviewed all the available laboratory data of the immune system from patient’s charts, including complete blood counts, immunoglobulin levels and distribution, T-cell recombination excision circles (TREC) and kappa-deleting element recombination circle (KREC) levels as well as T and B cell subpopulations. Information regarding various infections was extracted from the patient’s medical records and was further defined as viral, bacterial, fungal or parasitic. Quantification of TRECs and KRECs were determined by real-time quantitative (RQ)-PCR as was previously described [24, 25]. Age-matched healthy individuals were used as controls. Each experiment was performed in triplicate, and the threshold for Ct determination was positioned at the same level each time.

### Respiratory functions tests – Spirometry

In accordance with the AT clinic policy each patient had spirometry tests results recorded at least once. Lung function measurements including forced vital capacity (FVC %) and forced expiratory volume (FEV 1%) were recorded, and the best of three results was included in the study.

### Serum liver enzyme levels

Alanine transaminase (ALT) and aspartate aminotransferase (AST) levels were measured. Levels exceeding at least 1.5 times the upper limit of normal (ULN) on 3 or more separate occasions were considered abnormal, after extensive workup to exclude possible etiologies for LFT elevation.

### Imaging studies

Chest X ray, abdominal sonography and other imaging studies were performed as part of the ongoing care of the patients. All radiographs were obtained with computed radiography. All studies were evaluated by a pediatric radiologists on a PACS workstation (Easyvision, Sectra Imtec AB, Linköping, Sweden) using Totuko monitors (Totuko Electric co., ltd., Tokyo, Japan).

### Statistical analysis

All measured variables and derived parameters were tabulated by descriptive statistics. Pearson correlation coefficients were calculated for testing the relation between two continuous parameters. Student’s T-test was applied for testing the statistical significance of the difference between means in continuous variables. All tests applied were two-tailed, and a *p* value of 5% or less was considered statistically significant. Survival was assessed using the Kaplan-Meier method. Differences in survival outcomes were evaluated with the log-rank test. Statistical analysis was done using the IBM ™SPSS™ Version 21 software, New York, USA and Excel 2013 program.

## Results

Medical charts of 46 AT patients (24 males), age 14.6 ± 4.6 years, were reviewed. The average age of AT diagnosis was 4.3 ± 3.6 years, and average duration of follow-up was 8.3 ± 4 years (median 8 years, range 0.5–17 years). The diagnosis of AT was based on detection of ATM mutation in 35/46 (76%) of patients, low ATM on western blot in 9/46 (19.6%) and clinically in 2/46(4.4%) of patients.

Out of 46 patients with available IgM values, 15/46(33%) had EIgM with average serum IgM levels of 356 ± 200 mg/dl that were statistically higher than the average IgM levels seen in the NIgM group (129.2 ± 56.5 mg/dl, *p* < 0.001). Eight of 15 patients (53%) with EIgM, were males. There was no significant difference in average age between the EIgM group and the NIgM group (15.2 ± 2.3 years vs 14.4 ± 5.1 years, *p =* 0.32, respectively). Clinical and demographic characteristics of both groups are presented in Table 1.

**Table 1:** Clinical characteristics of EIgM and NIgM patients

Parameter^a^	EIgM	NIgM	p
N	15/46(32.6)	31/46(67.4)	N/A
Gender, male	8/15(53)	16/31(52)	*p =* 0.9
Average age, years	15.2 ± 2.8	14.3 ± 5.2	*p =* 0.35
Age of elevated IgM detection, years	9.3 ± 4.2	N/A	N/A
IgM levels (mg/dl)	356 ± 200	129.2 ± 56.5	*p < 0.001*
Deceased N/Total in the group	8/15(53.3)	9/31(29)	*p = 0.05*
Age at demise, years	13.7 ± 4.2	18.3 ± 6.3	*p = 0.042*

*N/A* Not Applicable

^a^Continuous variables are presented as mean ± SD. Categorical variables are presented as N (%)

### Complications

#### Infections

During the follow-up period, 88 infectious episodes were documented in all patients. Bacterial infections were the most frequent 66/88 (75%), followed by fungal 12/88 (13.6%), viral 9/88 (10.2%) and parasitic 1/88(1.2%) infections. AT patients suffered mainly from infections of the respiratory tract, as was previously described [18–20]. Chronic pulmonary colonization with *Mycobacterium gordonae* and *Pseudomonas aeruginosa* were significantly more frequent in the EIgM patients than in patients with NIgM, 4/15(27%) vs 1/31(3%), *p* < 0.017 and 7/15(47%) vs 6/31(19.4%), *p* = 0.05, respectfully. The incidence of infections with *Streptococcus pneumonia* was similar in both groups. Colonization of the lower respiratory tract with *Trichoderma sp., Candida sp. and Aspergillus niger* trended towards higher frequency in EIgM patients, but this trend did not reach statistical significance.

*Human Papilloma virus* skin infections were observed in 2 out of 15 EIgM patients (13.3%) while none were seen in the 31 NIgM patients (*p* = 0.04). However, skin infections with HSV and VZV were similarly frequent in both groups (1/15 vs 0/31patients with HSV skin infections and 2/15 vs 2/31 VZV infected patients, respectively). Systemic infections with *EBV* and *CMV* were rare and were only observed in 2 patients with NIgM. *Herpes simplex virus* type 1 (HSV1) encephalitis and severe disseminated infection with *Varicella zoster virus* (VZV) were seen in one patient NIgM and one patient with EIgM. Skin infection related to vaccinations were not observed.

#### Pulmonary disease

In light of the higher frequency of chronic pulmonary infection in the EIgM group, we expected a more severe pattern of chronic pulmonary disease in this group. Information regarding pulmonary functions was available for 23/31(74%) patients with NIgM, aged 12.2 ± 5.4 years and 10/15(67%) of patients with EIgM, aged 12.2 ± 2.5 years, at the time of observation. Average FEV1 and FVC measurements were similar between the EIgM and NIgM groups 51.1 ± 20.1 vs 41.8 ± 10.6, *p* = 0.12 and 42.7 ± 15.3 vs 36.5 ± 7.8, *p* = 0.13, respectively. During the follow up period, 329 chest X-ray examinations (CXR) were performed with an average of 1.2 ± 2.5 x-rays per patient per year of follow up in patients with NIgM vs 1.1 ± 2.1 x-rays per patient per year of follow up in patients with EIgM. All CXRs were re-evaluated by a pediatric radiologist for the presence of bronchiectasis in both groups, however, no significant difference was found. Bronchiectasis was present in 6 /31(19%) of the patients with NIgM compared to 3/15(20%) of the patients in the EIgM group, *p =* 0.87.

#### Liver function tests (LFTs)

LFTs were available in 41(89.1%) patients. There was no difference in the number of the EIgM and NIgM patients with elevated LFT (9/13(69%) vs13/28(46.4%), respectively, *p* = 0.2). Neither average AST nor ALT were significantly different between the groups (49.8 ± 10.6 vs 48.1 ± 27.8 IU/L, *p =* 0.4 and 33.7 ± 10 vs 47.2 ± 32.7 IU/L, *p* = 0.08, respectively). Abdominal sonography was performed in 14/15 (93.3%) patients with EIgM and 26/31(84%) with NIgM). Interestingly, none of the EIgM patients developed signs of fatty liver or cirrhosis, while 9 of the 26 (34.6%) patients with NIgM levels showed these liver abnormalities, *p* < 0.01. Average alpha-fetoprotein (AFP) levels were similarly elevated in both groups (286.5 ± 198 ng/ml in EIgM patients vs 251 ± 157 ng/ml in NIgM patients, *p* = 0.55).

#### Cancer

Cancer remained one of the main complications and leading causes of death among our patients. Three of 15 (20%) EIgM patients had cancer compared to 9 of 31(29%) patients with NIgM, *p =* 0.5. Lymphoma, acute lymphoblastic leukemia all of them T-cell (T-ALL) and solid tumors were the leading cancers seen in our cohort (8/46(17.4%), 4/46(8.7%) and 5/46(10.9%), respectively). These findings are similar to those reported in previously published cohorts [12, 26]. The average age of cancer diagnosis was 10.7 ± 3.3 years in the EIgM group vs 11.2 ± 6.2 years, in the NIgM patients. Several patients were diagnosed with more than one type of cancer.

#### Mortality and survival analysis

**Fig. 1:**
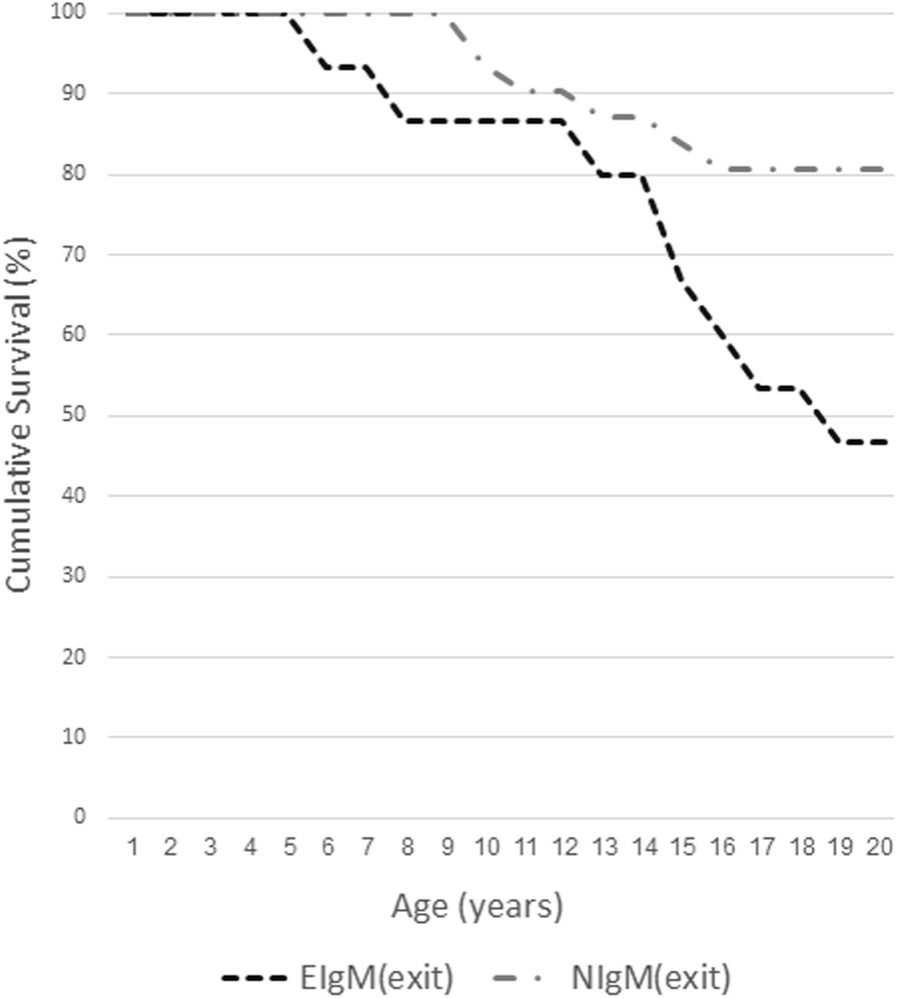
Kaplan-Meier curves for mortality in patients with NIgM levels and patients with EIgM. EIgM was associated with significantly increased mortality risk when using Log-Rank test to compare the 2 groups (z = 2.31, *p* = 0.05). The data is presented as the percent of patients alive at each age. The survival data is presented only for the first 20 years of life since only 7 patients from NIgM group and none of the EIgM group over 20 years of age were included in the study

During the 29 years of follow up 17(37%) patients died. The average age of death was 15.4 ± 6.3 for the entire cohort. The oldest patient alive at the time of the end of the study was 25.1 years old. The longest survival observed was 27.1 years. These results are similar to the survival observed in previously published cohorts [20, 26]. Patients with NIgM levels had significantly longer survival compared to patients with EIgM (z = 2.31, *p* = 0.05) (Fig. 1). The average age of death was 13.4 ± 4.2 years for the EIgM group vs 18.3 ± 6.3 years, (*p =* 0.042) in the NIgM patients.

#### Immunologic findings

As part of the routine follow-up of AT patients, a broad immunological evaluation was performed in most patients in our cohort. The baseline evaluation included complete blood count, immunoglobulin levels, T, B lymphocyte and NK cell sub-populations and basic complement studies. CD20+ lymphocytes and CD3+ lymphocytes were decreased in 8/46(17.4%) and 15/46(32.6%) of the patients, respectively, while 7/32(21.9%) of the patients had reduced lymphoproliferative responses. We did not observe any significant difference in lymphocyte subpopulations and lymphoproliferative responses between EIgM and NIgM patients. There was no significant difference in complement levels or in the ability to mount an adequate response to vaccination (data not shown).

The need for immunoglobulin replacement therapy with IVIG trended to be more frequent in the group of patients with EIgM in which 10/13(77%) received IVIG as compared to 14/27(52%), (*p =* 0.13) in the NIgM group.

Previous studies on this cohort of patients have demonstrated that AT patients have low T-cell recombination excision circles (TREC) and kappa-deleting element recombination circle (KREC) levels with abnormal TCR-Vβ repertoires [8].

Interestingly, despite the similar amounts of CD3+ and CD20+ cells in both groups, we observed a significantly lower KREK-CJ and TREK levels and a trend towards lower KREK-SJ levels in the EIgM group compared to the levels in normal IgM patients (Table 2).

**Table 2:** Immunological features of EIgM and NIgM patients

Parameter^a^	EIgM *N* = 15	NIgM *N* = 31	p
WBC (K/mL)	8.8 ± 1.6	8 ± 2.9	*p = 0.16*
ANC (K/ml)	5.6 ± 1.3	5.2 ± 2.5	*p = 0.3*
ALC (K/ml)	1.7 ± 0.5	1.9 ± 0.8	*p = 0.25*
CD3 + (K/ml)	1042 ± 526	959 ± 511	*p = 0.33*
CD4 + (K/ml)	466 ± 236	839 ± 1797	*p = 0.25*
CD8 + (K/ml)	597 ± 433	443 ± 247	*p = 0.09*
CD20 + (K/ml)	145 ± 67	167 ± 185	*p = 0.36*
CD56+ (K/ml)	26 ± 10	23 ± 10.7	*p = 0.24*
Patients treated with IVIG	10/13(77)	14/27(52)	*p = 0.13*
Age at IVIG initiation (years)	9.4 ± 5.7	6.6 ± 3.7	*p = 0.09*
Average IgA (mg/dL)	82 ± 97	59.5 ± 81	*p = 0.22*
C3(mg/dL)	146 ± 30.4	144.1 ± 11.3	*p = 0.46*
C4(mg/dL)	27 ± 8	37.4 ± 1.8	*p = 0.06*
KREC-CJ	732 ± 390	2568 ± 3115	*p = 0.033*
KREC-SJ	22 ± 17	112 ± 199	*p = 0.08*
TREC	13.9 ± 8.2	64.2 ± 93.5	*p = 0.046*
Normal lymphocyte proliferation	6/9(67)	19/23(83)	*p = 0.33*

*WBC* white blood cells, *ANC* Absolute neutrophil count, *ALC* absolute lymphocyte count, *IVIg* Intravenous immune globulin, *C* Complement, *NK* Natural Killer cell, *TREC* T cell receptor excision circle, *KREC* kappa-deleting recombination excision circles (*CJ* ψJα coding joint, *SJ* ψJα signal joint)

^a^Continuous variables are presented as mean ± SD. Categorical variables are presented as N (%)

## Discussion

AT is a rare, autosomal recessive, progressive, multi systemic disease with well described cellular and humoral immunodeficiency [4]. Hypogammaglobulinemia with low IgG, IgA and IgE levels, is a frequent presentation of this disease [27]. On the other hand, different studies have shown variability in IgM levels in these patients with normal or mildly elevated levels in up to 60% of the patients [19]. As a result, some AT patients are initially misdiagnosed with Hyper IgM syndrome, while the definitive AT diagnosis is made later by genetic analysis or western blot, following the accumulation of clinical characteristics indicative of AT. Recent studies advocated that elevated IgM levels should be included in the diagnostic criteria for AT suggesting that patients with this presentation might have a worse prognosis [19, 20].

In our cohort, 15 of 46 patients (32.6%) had elevated IgM levels, a slightly higher fraction as compared to previously published cohorts [19, 20, 28].

We have observed that AT patients with elevated IgM levels are more prone to colonization of the lower respiratory tract with pathogenic gram-negative bacteria as well as to higher frequency of viral skin infections, a presentation similar to that seen in other Hyper IgM phenotypes [29].

Despite these findings, we were not able to demonstrate a significant difference in pulmonary function between AT patients with normal or elevated IgM levels. This may have resulted from the fact that lung function testing in our cohort was done at a relatively young age, before recurrent lung infections had inflicted significant accumulative damage to the lung tissue.

Predisposition of AT patients to hepatic disease, which in some cases progresses to non-alcoholic steatohepatitis (NASH) and even cirrhosis with portal hypertension, was recently described [14]. In the current study, we observed that patients with elevated IgM levels did not present this phenotype. The progression of liver disease and insulin resistance in AT patients were recently shown to be age dependent [30]. Thus, it is possible that the shortened survival in the EIgM group paradoxically results in less apparent liver involvement, which might have developed, should these patients have survived longer. On the other hand, this could also be a distinctive clinical characteristic of the EIgM group. Further studies are needed to better illuminate this question.

Increased risk of developing cancer is a well-known feature of AT [12, 26, 31]. In contrast to previous reports, we did not observe an increased risk of malignancy in the group of EIgM patients.

Recent studies have investigated class switch recombination (CSR) as a critical mechanism in lymphocyte maturation in AT, reporting abnormal class switching in AT patients with high IgM levels [20, 28, 32]. The lower levels of KREC and TREC levels seen in the EIgM group in our cohort further supports these findings.

## Conclusions

Taken together, our findings further support previous reports suggesting that AT patients with elevated IgM levels represent a distinct group with a severe disease phenotype and worse prognosis resulting from a prominent CSR defect.

Further larger prospective studies in this subpopulation would be needed to establish a tailored management approach including close surveillance with early and aggressive treatment.

## Additional file

Additional file 1: **Table S1.** The reference range of immunoglobulin M (IgM) based on age*. (DOCX 13 kb)

## Abbreviations

AFP: Alfa fetoprotein
AT: Ataxia telangiectasia
ATM: Ataxia telangiectasia mutated
BMI: body mass index
CMV: Cytomegalovirus
CSR: Class switch recombination
EBV: Epstein-Barr virus
EIgM: Elevated IgM
FEV1: Forced expiratory volume (1 sec)
FVC: Forced vital capacity
HSV1: Herpes simplex virus type 1
IgM: Immunoglobulin M
IVIG: Intravenous immunoglobulin
KREC: Kappa recombination excision circles
NASH: Nonalcoholic steatohepatitis
NIgM: Normal IgM
NK: Natural killer cells
PEG: Percutaneous gastrostomy
TCR: T cell receptor
TREC: T-cell receptor excision circles
VZV: Varicella zoster virus
