# Supplementary material for: Elevated IgM levels as a marker for a unique phenotype in patients with Ataxia telangiectasia
Source: BMC Pediatr. 2018 Jun 4;18:185. doi: 10.1186/s12887-018-1156-1 (PMC5987459; doi:10.1186/s12887-018-1156-1)
Supplement: Supplementary file 1 — Table S1. The reference range of immunoglobulin M (IgM) based on age*. (DOCX 13 kb) [file 12887_2018_1156_MOESM1_ESM.docx]

**Table S1** The reference range of immunoglobulin M (IgM) based on age*:

| **Age** | **Range** |
| --- | --- |
| 0-1 years | 0-145mg/dL |
| 1-3 years | 19-146mg/dL |
| 4-6 years | 24-210 mg/dL |
| 7-9 years | 31-208 mg/dL |
| 10-11 years | 31-179 mg/dL |
| 12-13 years | 35-239 mg/dL |
| 14-15 years | 15-188mg/dL |
| 16-19 years | 23-259 mg/dL |
| >19 years | 40-230 mg/dL |

*****Copied from Medscape, Immunoglobulins, Rakesh Vadde, M Frances, J Schmidt, Eric B Staros.
